# Glycyl-tRNA Synthetase as a Target for Antiviral Drug Screening Against Influenza Virus

**DOI:** 10.3390/ijms26072912

**Published:** 2025-03-23

**Authors:** Jingjing Zhang, Xiaorong Li, Jingxian Liang, Xinru Meng, Chenchen Zhu, Guangpu Yang, Yali Liang, Qikai Zhou, Qianni Qin, Zan Li, Ting Zhang, Gen Liu, Litao Sun

**Affiliations:** 1 School of Public Health (Shenzhen), Shenzhen Campus of Sun Yat-sen University, Sun Yat-sen University, Shenzhen 518107, China; zhangjj75@mail2.sysu.edu.cn (J.Z.); lixr36@mail2.sysu.edu.cn (X.L.); liangjx37@mail2.sysu.edu.cn (J.L.); mengxr6@mail2.sysu.edu.cn (X.M.); yangguangpu183@163.com (G.Y.); liangyali2023@163.com (Y.L.); qinqn@mail2.sysu.edu.cn (Q.Q.); lizan6@mail2.sysu.edu.cn (Z.L.); 2 Shenzhen Key Laboratory of Pathogenic Microbes and Biosafety, Shenzhen 518107, China; 3 School of Medicine, Shenzhen Campus of Sun Yat-sen University, Sun Yat-sen University, Shenzhen 518107, China; zcc099288@163.com (C.Z.); zhangt377@mail2.sysu.edu.cn (T.Z.); 4 School of Biomedical Engineering, Med-X Research Institute, Shanghai Jiao Tong University, Shanghai 200030, China; simon-zhou@sjtu.edu.cn

**Keywords:** Glycyl-tRNA synthetase, influenza virus, antiviral drug screening

## Abstract

Influenza viruses are characterized by their high variability and pathogenicity, and effective therapeutic options remain limited. Given these challenges, targeting host cell proteins that facilitate viral replication presents a promising strategy for antiviral drug discovery. In the present study, we observed a significant upregulation of Glycyl-tRNA synthetase (GlyRS) within 24 h post-PR8 virus infection. The inhibition of GlyRS expression in A549 cells resulted in a marked reduction in infection rates across multiple influenza virus strains, while the overexpression of GlyRS led to an increase in viral infectivity during the early stages of infection. These findings suggest that GlyRS plays a critical role in the replication of influenza virus. Accordingly, we screened for potential inhibitors targeting GlyRS and identified Lycobetaine and Scutellarein using a multifaceted approach. Through a combination of molecular dynamics simulations, we further elucidated the mechanisms of action and potential binding sites of these compounds. Both inhibitors effectively suppressed the replication of influenza viruses, and their antiviral activity was confirmed to be mediated by GlyRS targeting. Therefore, GlyRS inhibitors, such as Lycobetaine and Scutellarein, represent promising candidates for combating influenza infections and provide novel insights into the treatment of influenza and aaRS-related diseases, opening new avenues for the development of aaRS-targeted therapeutics.

## 1. Introduction

Influenza viruses with a negative-sense genome have the potential to cause pandemics. Vaccines are available for influenza but antiviral drugs are still important for those infected with different strains and the unvaccinated [1]. New antiviral drugs are important due to the potential for emergence of drug resistance, as seen for amantadine [2]. As obligate parasites, viruses depend on host factors for efficient replication. Targeting host factors that are essential to the viral life cycle offers an ideal therapeutic strategy to prevent the development of drug resistance [3]. This strategy, which focuses on host proteins rather than directly targeting the virus itself, can effectively reduce the risk of drug resistance due to viral mutations and can provide broad-spectrum antiviral effects against different viruses [4]. Previous studies have indicated that the heat shock protein 90 (HSP90) chaperone may play a role in SARS-CoV-2 replication and COVID-19 pathogenesis, with pharmacological interventions using HSP90 inhibitor, Ganetespib (STA-9090), shown to reduce viral replication across various viruses [5]. E-64d, an inhibitor of Cathepsin L (CTSL), can block SARS-CoV-2 pseudovirus infection and has demonstrated potential in the treatment of related viral infections [6]. This indicates that small molecule inhibitors targeting host intracellular enzymes were identified and gained attention for their potential in antiviral therapy.

In this regard, the activity of human aminoacyl-tRNA synthetases (aaRSs) are noteworthy. Viruses, as obligate intracellular parasites with minimal genetic information, rely on hijacking host cellular resources, including aaRSs and other proteins, to facilitate the translation of viral mRNA [7]. aaRSs are essential enzymes that covalently link substrate amino acids to homologous tRNA for protein synthesis, and they also act as regulators of cellular processes by sensing various cellular conditions [8]. Non- multi-tRNA synthetase complex (MSC) cytoplasmic aaRSs, along with mitochondrial Lysyl-tRNA Synthetase 2 (KARS2), play a role in supporting viral life cycles [9]. Similarly, Tryptophanyl-tRNA synthetase 1 (WARS1) serves as an IFN-γ-inducible mediator of enterovirus cell entry, such as EV-A71, and acts as a cell type-specific restriction factor [9]. Tryptophanyl-tRNA synthetase 2 (WARS2) positively regulates pseudorabies virus (PRV) proliferation [10].

The GARS gene encodes GlyRS (Glycyl-tRNA synthetase), a member of the aaRS family, a bifunctional enzyme responsible for tRNA charging, which can be localized either to the cytoplasm or mitochondria [11]. Disease-associated GARS mutations are involved in neurotoxicity in Charcot–Marie–Tooth disease (CMT) [12] and GARS was reported to be associated with the occurrence and development of various cancers [13,14,15]. The precise role of GARS in viral infections has not been extensively explored. However, given GARS’s critical function and significance across various diseases, its essential roles suggest that it may also have potential involvement in viral pathogenesis.

In this study, we first observed a significant increase in GlyRS levels 24 h after PR8 viral infection, indicating that GlyRS may play a crucial role in the viral infection process. Through further experiments, we confirmed that reducing GlyRS expression effectively lowered the viral infection rates. Additionally, we tested various influenza virus strains beyond PR8, indicating that GlyRS levels are closely associated with influenza virus replication and dissemination. Subsequently, we conducted an in vitro antiviral drug screening targeting GlyRS. We screened 5090 bioactive compounds and identified Lycobetaine and Scutellarein as effective inhibitors of GlyRS with significant antiviral activity. This finding not only reveals the potential of these compounds as GlyRS inhibitors but also provides new candidate molecules for antiviral drugs targeting GlyRS. Our results further support the potential of GlyRS as a therapeutic target in viral infections. The important role of GlyRS in viral infections makes it a novel target for intervening in viral replication.

## 2. Results

### 2.1. GlyRS Is Associated with the Proliferation of the PR8 Virus

**Figure 1:**
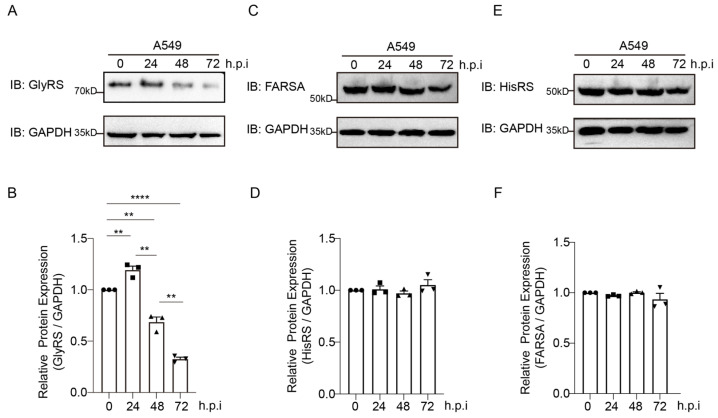
Changes in Aminoacyl-tRNA synthetase expression post Influenza A Virus Infection. The changes in GlyRS (**A**), FARSA (**C**), and HisRS (**E**) expression were measured at 24, 48, and 72 h post-viral infection by Western blot. The experiment was repeated three times, and the results presented here are from one representative experiment. The results from the three repeated experiments were quantified using ImageJ in (**B**,**D**,**F**). Compared and analyzed using a *t*-test. **: *p* < 0.01, ****: *p* < 0.0001.

To evaluate the relationship between GlyRS and viral infection, we infected A549 cells with PR8 virus at a MOI (multiplicity of infection) of 1 and assessed the effect of viral infection on GlyRS expression using Western blot at 24, 48, and 72 h post-infection (Figure 1A). The experiment was repeated three times, and changes were analyzed using ImageJ 1.54f. We observed a significant increase in GlyRS expression at 24 h post-infection, which then gradually decreased after 24 h (Figure 1B). Phenylalanine-tRNA synthetase (FARS) subunit alpha (FARSA) encodes one of the two alpha subunits of FARS, a typically heterotetrameric protein composed of two alpha and two beta subunits, the latter encoded by the FARSB gene [16]. Histidyl-tRNA synthetase (HisRS or HARS1) and GlyRS are associated with CMT disease, with HisRS classified as subtype CMT2W [17]. We also examined the changes in these two aminoacyl-tRNA synthetases during viral infection. Our results indicate that the expression levels of these two aminoacyl-tRNA synthetases do not show significant changes during viral infection (Figure 1C–F). This phenomenon indicates that GlyRS is involved in viral infection and may play a significant role in the process.

**Figure 2:**
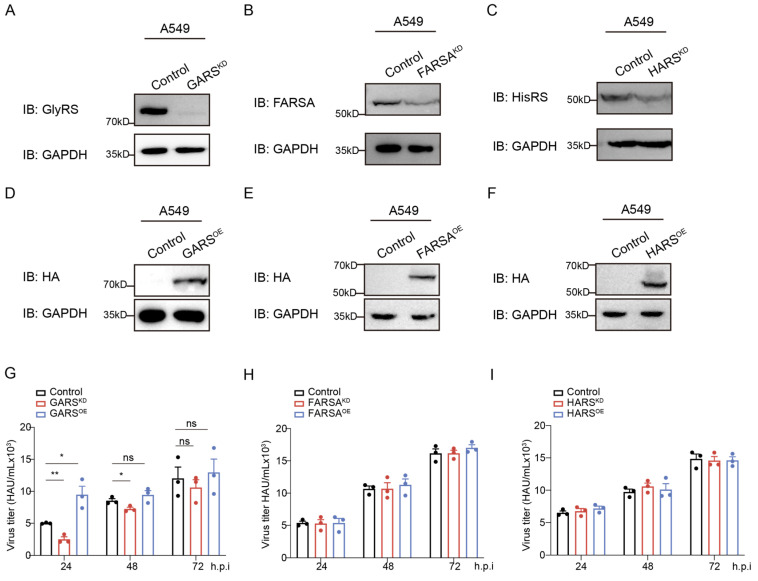
Generation of aaRS^KD^ cells and the role of GARS in influenza A virus infection. Stable cell lines were established by infecting A549 cells with packaged lentivirus, and the knockdown efficiency of GARS (**A**), FARSA (**B**), and HisRS (**C**) in puromycin-selected A549 cells was assessed through immunoblot analysis. In A549 cells, Western blot analysis was conducted 48 h post-transient transfection with HA-GARS (**D**), HA-FARSA (**E**), HA-HisRS (**F**), and HA-vector, utilizing HA antibodies for detection. (**G**) GARS^KD^, GARS^OE^ (transient transfection of HA-GARS), or negative control A549 cells were infected with the PR8 virus at a MOI of 1. (**H**) FARSA^KD^, FARSA^OE^ (transient transfection of HA-FARSA), or negative control A549 cells were infected with the PR8 virus at a MOI of 1. (**I**) HisRS^KD^, HisRS^OE^ (transient transfection of HA-HisRS), or negative control A549 cells were infected with the PR8 virus at a MOI of 1. The virus titers were measured using the hemagglutination method at 24, 48, and 72 h post-viral infection. Data are presented as the mean of three independent experiments, with error bars representing the standard error of the mean (SEM). ns: not significant, *: *p* < 0.05, **: *p* < 0.01.

To validate this hypothesis, we constructed knockdown plasmids and used 293T cells for lentivirus packaging. The obtained virus was used to infect A549 cells, followed by puromycin selection until GlyRS knockdown was confirmed (Figure 2A). We successfully established stable cell lines, laying the foundation for further research on the effect of this gene on viral infection. Meanwhile, we also created knockdown cell lines for FARSA (Figure 2B) and HARS (Figure 2C). We then transiently expressed HA-tagged HA-GlyRS, HA-FARSA, and HA-HisRS in A549 cells and confirmed successful expression (Figure 2D–F).

Next, we infected these aaRS knockdown cell lines and aaRS overexpression cells with PR8 virus to determine the effect of aaRS expression on viral infection. Surprisingly, we found that GlyRS knockdown effectively inhibited viral replication, while GlyRS overexpression promoted viral replication. This effect was most pronounced 24 h post-infection (Figure 2G). However, the expression levels of FARSA or HARS did not have a significant impact on viral titers (Figure 2H,I). The significant inhibition of viral replication observed 24 h after GlyRS knockdown indicates that GlyRS plays a crucial role in the early stages of viral infection. Compared to other aaRSs, the specificity and function of GlyRS during viral infection also provide directions for future research.

### 2.2. Knockdown of GlyRS Decreases Infection of Multiple Influenza Virus Strains

**Figure 3:**
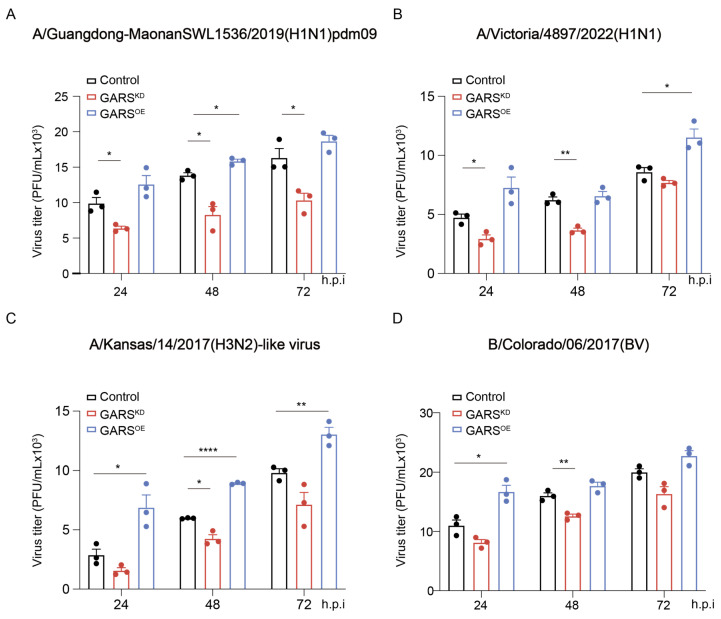
A549 cells were infected with A/Guangdong-MaonanSWL1536/2019(H1N1)pdm09 virus (**A**) at an MOI of 1, A/Victoria/4897/2022(H1N1) (**B**) at an MOI of 0.5, A/Kansas/14/2017(H3N2)-like virus (**C**) at an MOI of 0.5, and B/Colorado/06/2017(BV) (**D**) at an MOI of 0.1. Virus titers were measured using the hemagglutination method at 24, 48, and 72 h post-infection. Data are presented as the mean of three independent experiments, with error bars representing the standard error of the mean (SEM). ns: not significant, *: *p* < 0.05, **: *p* < 0.01, ****: *p* < 0.0001.

To further verify the broad-spectrum potential of GlyRS in influenza viruses, we also selected other influenza virus strains for infection experiments, including different subtypes of type A, such as H1N1 and H3N2, as well as type B (Figure 3A–D). By measuring the viral titers and infection rates of these different strains, we found that GlyRS played a similar role in the replication process of these influenza viruses. Whether it was H1N1, H3N2, or type B influenza virus, inhibiting GlyRS expression resulted in a significant reduction in viral replication and spread, while the overexpression of GlyRS promoted viral replication. These results further demonstrate that GlyRS plays a broad role in the influenza virus life cycle, not limited to a specific viral strain. Therefore, the potential of GlyRS as an antiviral target may have application value for multiple influenza virus subtypes, offering new insights for the development of broad-spectrum antiviral drugs against influenza.

We also selected Zika virus as a control for comparison and conducted the relevant experiments. The results showed that the expression level of GlyRS had no significant effect on the expression of NS5 protein two days after Zika virus infection (Figure S1). This finding indicates that the antiviral effect of GlyRS on influenza virus is specific, and it does not exert the same effect on other viruses, such as Zika virus. This result further supports our hypothesis of GlyRS as a target for influenza virus.

### 2.3. Multi-Approach Drug Screening Based on GlyRS

**Figure 4:**
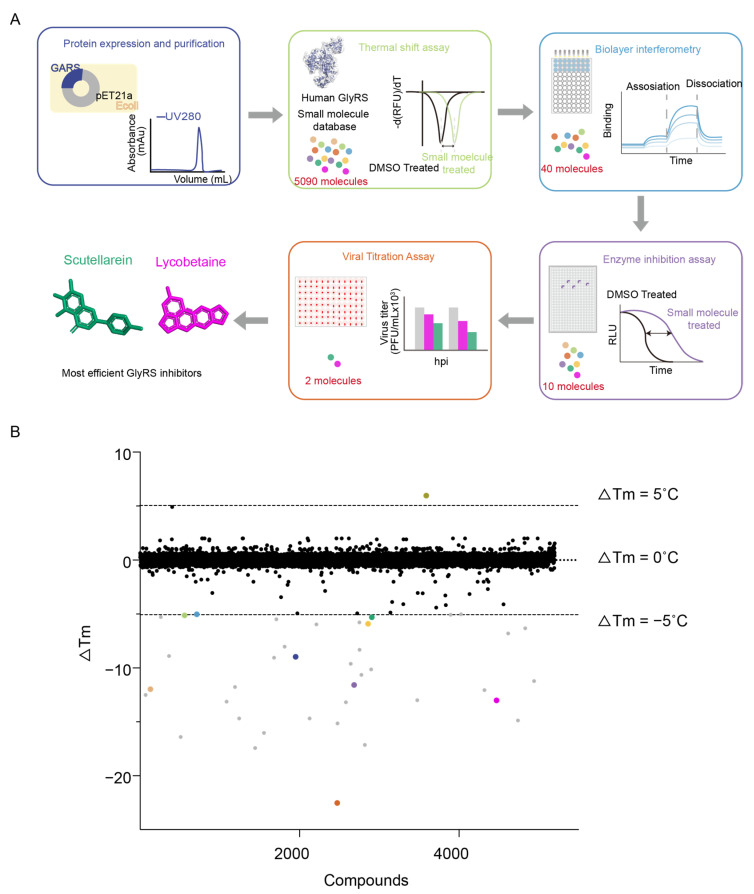
Preliminary screening of antiviral drugs through GlyRS. (**A**) Flow chart of antiviral drug screening through GlyRS. Drug screening strategy includes thermal shift assay, bio-layer interferometry, enzyme inhibition assay, and viral titration assay. (**B**) Out of 5090 compounds, 40 exhibited a Tm shift > 5.0 °C, indicating positive hits. Black points within the black dashed lines represent negative results, while gray and colored points outside the gray dashed lines indicate positive hits.

**Figure 5:**
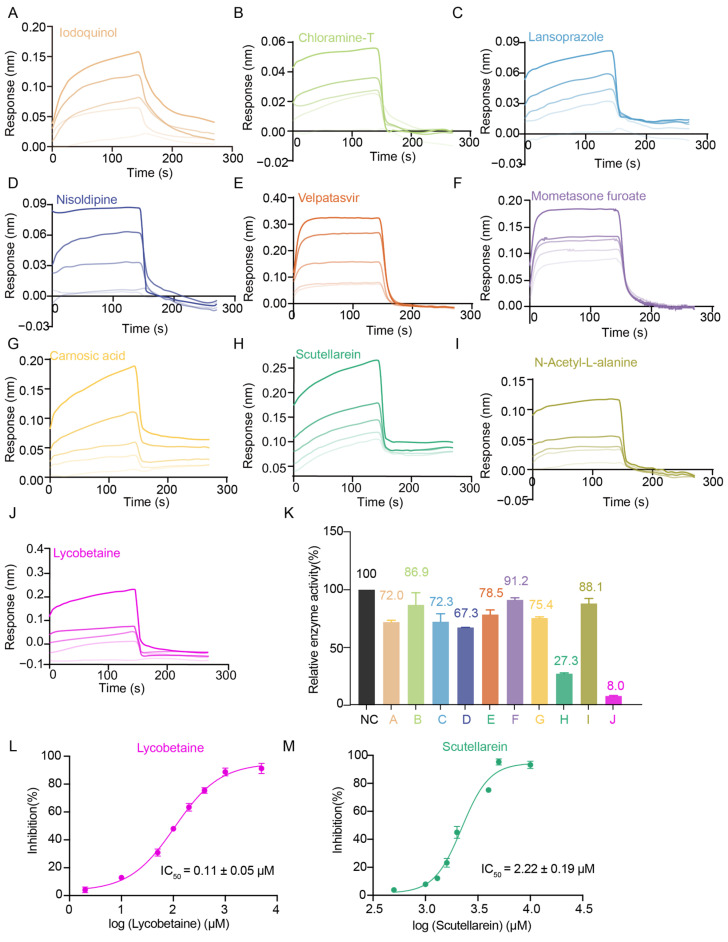
The identification of GlyRS inhibitors. Binding sensorgrams for the interaction of Iodoquiol (**A**), Chloramine-T (**B**), Lansoprazole (**C**), Nisoldipine (**D**), Velpatasvir (**E**), Mometasone furoate (**F**), Carnosic acid (**G**), Scutellarein (**H**), N-Acetyl-L-alanine (**I**), and Lycobetaine (**J**) with immobilized GlyRS. The five curves are generated from 20, 10, 5, 2.5, and 1.25 μM molecules from top to bottom. The BLI affinity of 10 molecules to GlyRS was presented as mean ± SD, as shown in Table 1. The experiment was repeated three times, and the result shown in the image is a representative outcome of one of these repetitions. (**K**) The impact of 10 small molecules on GlyRS enzyme activity. The IC_50_ values of Lycobetaine (**L**) or Scutellarein (**M**) were measured based on the ATP consumption assay and presented as mean ± SD.

Next, we aimed at screening drugs targeting GlyRS to identify those that can inhibit its function, with the goal of advancing antiviral treatment. To identify the potential molecules targeting GlyRS, we performed a series of different experiments including thermal shift assay (TSA), bio-layer interferometry (BLI), enzyme inhibition assay, and viral titration assay (Figure 4A). Initially, the recombinant GlyRS protein was purified and utilized in a fluorescence-based thermal shift assay. We screened 5090 small molecules (Table S1), including 2734 bioactive compounds and 2356 FDA-approved pharmaceuticals, and identified 40 hits with Tm shifts > 5.0 °C (Figure 4B). For further validation, we conducted a BLI assay to analyze the binding kinetics and affinity between the small molecules identified in TSA and GlyRS. We identified 10 hits that interacted with GlyRS and measured their affinity (K_D_); the specific BLI binding sensorgrams for these 10 drugs are also displayed in the table and figure (Table 1, Figure 5A–J). To investigate whether these molecules affect the aminoacylation activity of GlyRS, we performed enzyme assays and revealed that only two compounds named Lycobetaine and Scutellarein interacted with GlyRS and inhibited its aminoacylation (Table 2, Figure 5K). To quantify the inhibitory activity of Lycobetaine and Scutellarein on GlyRS, we measured their IC_50_ values by enzyme inhibition assay. The IC_50_ value for Lycobetaine was determined to be 0.11 ± 0.05 μM, while for Scutellarein, it was 2.22 ± 0.19 μM (Figure 5L,M). These results demonstrate the potency of Lycobetaine and Scutellarein in inhibiting GlyRS function.

### 2.4. Binding Mode of Screened Inhibitors with GlyRS

**Figure 6:**
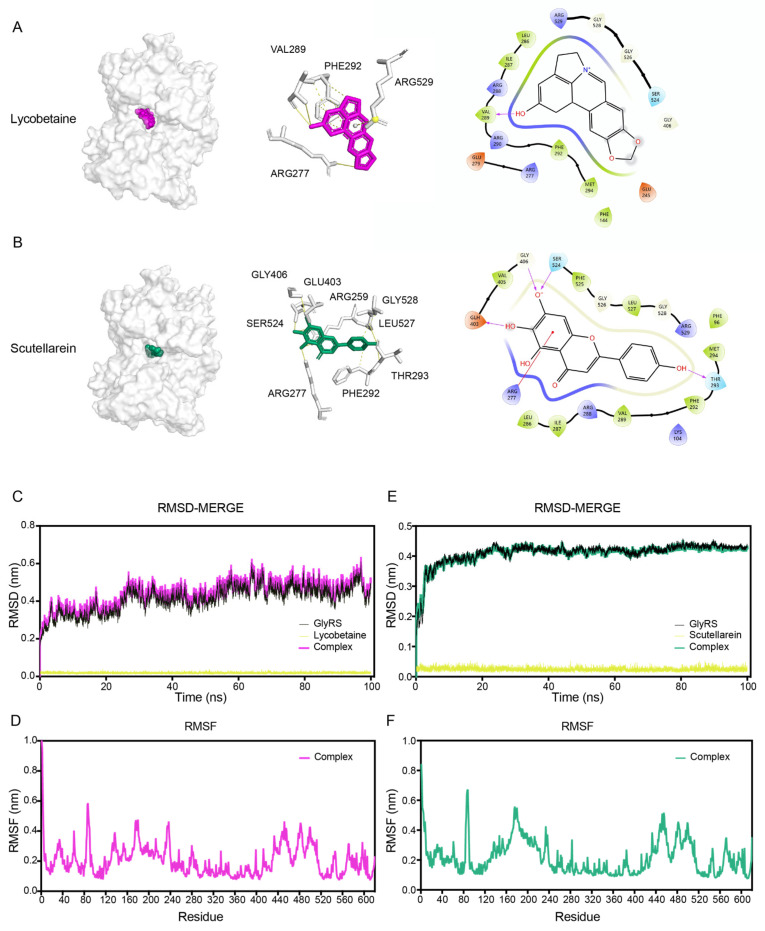
Binding modes and molecular dynamics simulations of inhibitors with GlyRS. The binding pocket and site of Lycobetaine (**A**) or Scutellarein (**B**) and the GlyRS protein and its interaction with surrounding amino acids. The two-dimensional plot was generated using Schrödinger software (Release 2019-2, Schrödinger LLC, New York, NY, USA, 2019). The curve of RMSD values over time during the molecular dynamics simulation of the Lycobetaine (**C**) or Scutellarein (**D**) and GlyRS complex. The MD simulation (RMSF analysis) of GlyRS and Lycobetaine (**E**) or Scutellarein (**F**) complexes for 100 ns.

To further confirm the binding modes of Lycobetaine and Scutellarein with GlyRS, we conducted molecular docking and also performed docking studies on the remaining eight small molecules. Based on the docking scores, Lycobetaine and Scutellarein exhibited favorable binding activity with GlyRS among these small molecules (Table 3). Lycobetaine demonstrated compatibility with the active pocket of the GlyRS target and could establish binding within the GlyRS active pocket, forming hydrogen bonds with ARG277, VAL289, PHE292, and ARG529 and the docking score was −8.587 (Figure 6A). The docking score for the interaction between Scutellarein and GlyRS was −8.573, and Scutellarein could interact with GlyRS at the same pocket by forming hydrogen bonds with ARG259, ARG277, PHE292, THR293, GLU403, GLY406, SER524, LEU527, and GLY528 (Figure 6B). To further investigate the stability of the binding conformation between Lycobetaine or Scutellarein and GlyRS, molecular dynamics simulations were employed. The simulation lasted for 100 ns to analyze the stability and rigidity of the protein–ligand complexes. The stability of the entire complex was analyzed based on the root-mean-square deviation (RMSD). A root-mean-square fluctuation (RMSF) analysis was used to analyze the conformational fluctuation of the complex. The average RMSD of the complex formed by Lycobetaine and GlyRS was <0.4 nm and began to approach equilibrium after 30 ns (Figure 6C). The RMSF analysis of the Lycobetaine and GlyRS complex indicated minimal fluctuations in the majority of amino acids, ranging from 0.1 nm to 0.5 nm (Figure 6D). The average RMSD of the complex formed by Scutellarein and GlyRS was <0.5 nm and began to approach equilibrium after 10 ns (Figure 6E). The RMSF analysis of the Scutellarein and GlyRS complex indicated minimal fluctuations in the majority of amino acids, ranging from 0.1 nm to 0.6 nm (Figure 6F). The results indicate that both Lycobetaine and Scutellarein maintain good conformational stability in the GlyRS complexes throughout the simulation process.

### 2.5. GlyRS Inhibitors Can Block Influenza Virus

**Figure 7:**
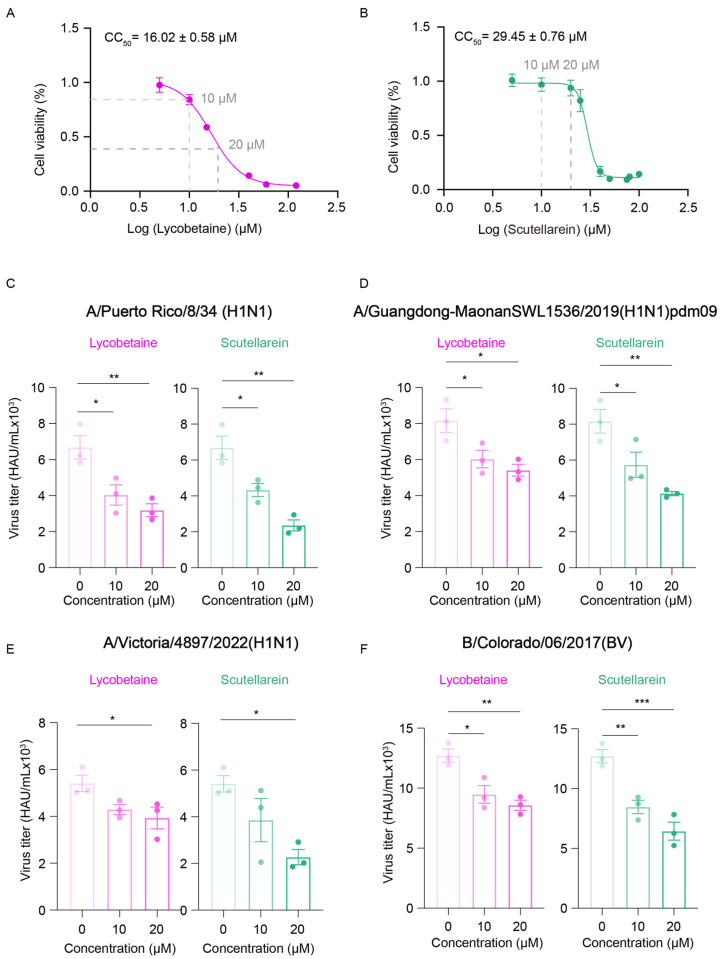
GlyRS inhibitors block virus infection. The CCK-8 assay revealed the CC_50_ value of Lycobetaine (**A**) or Scutellarein (**B**) on A549 cells. Data were presented as mean ± SD. The vertical markers indicate the positions of the drugs at 10 μM or 20 μM. After inhibitor treatment for 24h and during viral infection, A/PuertoRico/8/34 (H1N1) (**C**) and A/Guangdong-MaonanSWL1536/2019 (H1N1) pdm09 virus (**D**) at a MOI of 1, A/Victoria/4897/2022(H1N1) (**E**) at a MOI of 0.5, and B/Colorado/06/2017(BV) (**F**) at a MOI of 0.1 were used to infect A549 cells. The virus titers were measured using the hemagglutination method at 24 h post-viral infection. *: *p* < 0.05, **: *p* < 0.01, ***: *p* < 0.001.

We then evaluated the antiviral effects of Lycobetaine and Scutellarein. To determine the optimal concentration of the small molecules, we used the CCK-8 assay to evaluate cell viability and estimate the CC_50_ value of the two drugs in A549 cells [18]. In this experiment, the cells were exposed to varying concentrations of Lycobetaine and Scutellarein, and the CCK-8 reagent was added 24 h after drug treatment. By measuring the color intensity, we assessed cell viability and calculated the CC_50_ values using these data. We measured the CC_50_ values of Lycobetaine and Scutellarein for the inhibition of A549 cells, which were 16.02 ± 0.58 μM and 29.45 ± 0.76 μM, respectively (Figure 7A,B).

To assess whether GlyRS inhibitors have antiviral effects in the early stages of infection, we treated A549 cells with 10 μM or 20 μM Lycobetaine and Scutellarein for 24 h before PR8 infection and measured viral titer 24 h post-PR8 infection. The results indicate that the drug treatments remained during viral infection. We found that both GlyRS inhibitors effectively reduced the viral infection rate at 24 h post-infection (Figure 7C). This indicates that screening for small molecule lead compounds that inhibit GlyRS function as potential antiviral drugs is feasible. We also tested the antiviral effects of Lycobetaine and Scutellarein against other influenza virus strains to assess their broad-spectrum activity. In addition to the PR8 virus, we included other H1N1 strains and B-type influenza viruses (Figure 7D–F). Both drugs consistently inhibited viral replication and spread across all tested strains, demonstrating their broad-spectrum antiviral activity. Specifically, Lycobetaine and Scutellarein reduced viral titers and infection rates for both A-type and B-type influenza viruses, confirming GlyRS as an effective target for antiviral therapy and providing new insights into the development of broad-spectrum antiviral drugs. We evaluated the antiviral Selectivity Index (SI) of both Lycobetaine and Scutellarein (Figures S2 and S3). The results showed that Lycobetaine had an SI value for influenza viruses ranging from approximately 0.5 to 2, indicating a certain level of antiviral activity with minimal cytotoxic effects at appropriate concentrations. Meanwhile, Scutellarein exhibited an SI value of around 2 for influenza viruses, demonstrating more significant antiviral effects and a better balance between cytotoxicity and antiviral activity. The IC50 value obtained by pmd09 for the inhibition test using zanamivir as a positive control (Figure S4) was similar to that reported in previous studies [19], suggesting that the experimental system was working properly.

### 2.6. Lycobetaine Alters Conformation of GlyRS and Blocks Virus by Targeting GlyRS

Although both small molecules form stable complexes with GlyRS, it remains uncertain whether they induce conformational changes in specific regions of the enzyme. Such alterations could potentially disrupt GlyRS’s normal function, affecting its catalytic activity or interactions with other molecules. To more clearly elucidate the mechanisms of action of the two inhibitors on GlyRS, we employed limited proteolysis analysis to study protein structure and conformational changes. This method involves treating the protein or its complexes with different concentrations of trypsin at certain temperatures, causing cleavage at primarily exposed/surface arginine or lysine sites. When small molecules alter the protein’s conformation, the distribution of the proteolyzed protein fragments will be significantly different.

**Figure 8:**
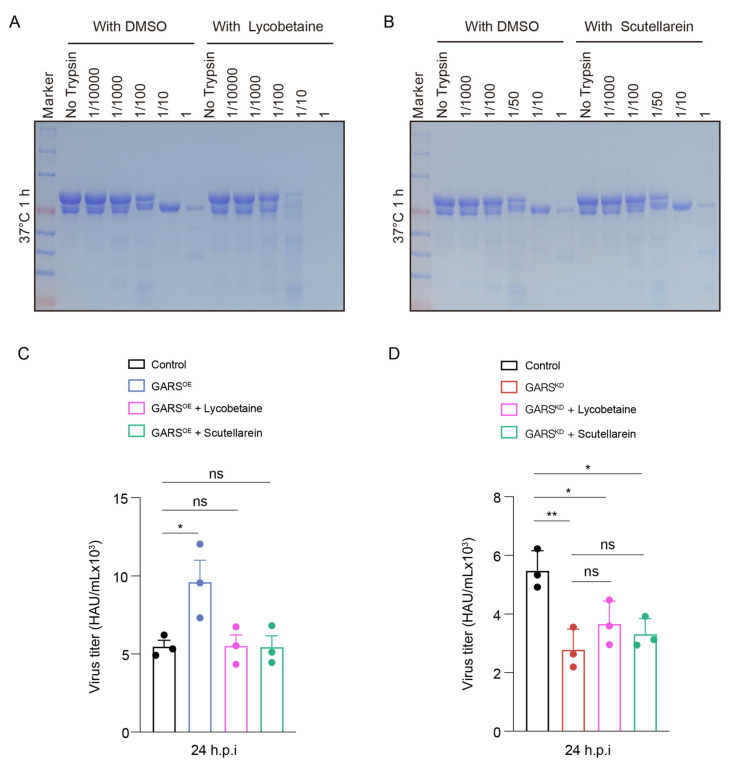
Lycobetaine induces conformational changes in GlyRS and inhibits virus infection by GlyRS. In the limited proteolysis analysis, the GlyRS proteins (in the presence and absence of Lycobetaine (**A**) or Scutellarein (**B**)) were incubated with trypsin at different concentrations for 1 h at 37 °C before the reactions were quenched, and the products were separated by SDS-PAGE. (**C**) After inhibitor treatment for 24h and PR8 infection at a MOI of 1 on GARS^OE^ A549 cells, with the inhibitor present throughout the entire treatment. The virus titers were measured using the hemagglutination method at 24 h post-viral infection. *: *p* < 0.05. (**D**) After inhibitor treatment for 24 h and PR8 infection at a MOI of 1 on GARS^KD^ A549 cells, with the inhibitor present throughout the entire treatment. The virus titers were measured using the hemagglutination method at 24 h post-viral infection. *: *p* < 0.05, **: *p* < 0.01. ns: none significance.

Our results indicated that, compared to unbound GlyRS, GlyRS bound to Lycobetaine is digested at lower concentrations after one hour of proteolysis at 37 °C (Figure 8A). Additionally, the distribution of the proteolyzed protein fragments is significantly different from that of unbound GlyRS, suggesting that Lycobetaine alters the protein conformation of GlyRS. This also suggests that Lycobetaine may affect its non-canonical function by altering the conformation of GlyRS. However, when we performed proteolysis experiments with GlyRS bound to Scutellarein, we found that it did not show significant differences compared to unbounded GlyRS (Figure 8B). This suggests that not all GlyRS inhibitors need to alter the protein conformation of GlyRS. Instead, as long as they impact its functional activity, they can effectively exert antiviral effects.

To verify whether the antiviral efficacy of these two drugs is mediated through GlyRS, we treated GlyRS overexpression cells and GlyRS knockdown cells with the two drugs and measured the viral titer 24 h post-PR8 infection. We observed that overexpression of GlyRS during viral infection promotes viral infection at 24 h and can counteract the antiviral effects induced by the drugs (Figure 8C). This indicates that GlyRS is indeed a target for the antiviral action of these two drugs within the cell, and the antiviral effects of both small molecules are mediated through the inhibition of GlyRS function. However, adding the two drugs to the GlyRS knockdown stable cell lines did not significantly affect the viral titer, further indicating that the antiviral effects of GlyRS inhibitors are dependent on the presence of GlyRS (Figure 8D).

In summary, we identified two inhibitors of GlyRS. Lycobetaine induces conformational changes in the GlyRS protein, while Scutellarein does not affect protein conformation. Nonetheless, both inhibitors demonstrate antiviral activity within the cells.

## 3. Discussion

aaRSs are vital enzymes that play a crucial role in protein synthesis. They achieve this by ligating amino acids to their corresponding tRNAs, forming aminoacylated-tRNAs, which are essential for decoding genetic information during protein translation [20,21,22]. Various aaRSs are also involved in a wide range of physiological and pathological processes, including the regulation of angiogenesis, inflammation, cancer, and neurodegenerative diseases [23,24,25,26,27,28]. Due to the multifaceted functions of aaRSs, inhibitors of aaRSs are utilized as lead compounds in disease treatments across various fields, including antimicrobial, antimalarial, and anticancer therapies [29,30,31,32]. Although human aaRSs are considered unsuitable targets due to their critical role in protein synthesis, studies have shown that normal cells can tolerate aaRS inhibition [31]. Moreover, the development of aaRS-specific compounds will not only provide potential therapies for treating these human diseases but also offer unprecedented tools to directly dissect the molecular mechanisms of aaRS-related pathogenesis and their unknown biological functions [33].

The GlyRS inhibitor we identified, the betaine-type phenanthridine alkaloid Lycobetaine, is a minor constituent of the Amaryllidaceae family [34]. Its derivatives were reported to possess antiviral and anticancer effects [35,36]. The specific inhibitory mechanism of Lycobetaine and its derivatives has not been determined in previous studies [37]. The interaction between Lycobetaine and GlyRS provides new insights into the antiviral mechanism of Lycobetaine and its derivatives. Pharmacological studies reveal that Scutellarein exhibits a broad spectrum of pharmacological properties, including but not limited to anti-inflammatory, antioxidant, antiviral, neuroprotective, hypoglycemic, hypolipidemic, anticancer, and cardiovascular protective effects [38]. These pharmacological studies suggest that Scutellarein holds promise for treating a variety of diseases due to its diverse range of effects. Its influence on GlyRS function opens new avenues for understanding how Scutellarein may act within different therapeutic contexts. By elucidating how Scutellarein modulates GlyRS, researchers can gain deeper insights into its potential mechanisms of action across different disease models, potentially uncovering novel therapeutic strategies and optimizing its use in clinical applications.

Although our study has yielded significant findings, there are still limitations that need to be addressed. Firstly, while Lycobetaine and Scutellarein demonstrated strong binding with GlyRS in vitro and exhibited inhibitory effects on influenza virus infection, further validation in animal models is required to confirm the therapeutic efficacy of the drug. Secondly, aaRSs regulate many functions in cells through non-canonical pathways [39]. Therefore, the non-classical mechanism by which Lycobetaine and Scutellarein inhibit GlyRS function to impact viral infection requires further elucidation. Lycobetine and Scutellarein, despite their current low SI values, demonstrate clear target specificity and antiviral activity at subtoxic concentrations, as evidenced by GlyRS-targeting experiments and viral titer inhibition assays. These findings highlight their potential as lead compounds for further optimization. By leveraging structure–activity relationship (SAR) studies and rational drug design, it is possible to enhance their selectivity, reduce off-target effects, and improve their overall therapeutic profiles. Future research could focus on elucidating the specific mechanisms of GlyRS in viral infections and optimizing the drug properties and clinical application potential of Lycobetaine and Scutellarein. Through these efforts, we aim to offer new strategies and effective treatment options for antiviral therapy.

## 4. Materials and Methods

### 4.1. Cell Lines and Cell Culture

A549 and 293T cells were cultured in Dulbecco’s Modified Eagle Medium (DMEM, Gibco, Carlsbad, CA, USA) supplemented with 1% penicillin/streptomycin and 10% FBS (FBS, Gibco, Carlsbad, CA, USA). All cell lines used in this study were incubated at 37 °C in a humidified incubator containing 5% CO_2_.

### 4.2. Influenza Virus Infection

First, the cell culture medium was discarded to ensure no residual medium remained. The cells were then thoroughly washed twice with sterile PBS to remove any remaining medium or impurities. After washing, the influenza virus was diluted according to the required MOI for the experiment, ensuring the viral suspension was evenly distributed. The viral suspension was added to the cell culture dish and incubated at 37 °C for 2 h, allowing the virus to fully bind with the cells and initiate infection. After the infection period, the viral suspension was removed, and the cells were washed twice again with PBS to eliminate any unbound viral particles. The cells were then transferred to DMEM medium containing 0.5 μg/mL TPCK (tosylsulfonyl phenylalanyl chloromethyl ketone)-treated trypsin and 2% FBS for further cultivation. The TPCK-treated trypsin helps facilitate viral maturation and activates its infectivity, while 2% FBS provides essential nutrients to maintain cell stability post-infection, allowing for subsequent analysis and experiments.

### 4.3. Virus Titer Detection

To determine the virus titer, viral supernatants were collected, and virus titers were determined by the hemagglutination test. In this hemagglutination assay, 1% turkey red blood cell (RBC) suspension is prepared by washing RBCs stored in Alsever’s solution with PBS, centrifuging at 1500 rpm, and repeating this process three times before resuspending in PBS. The test virus is serially diluted in a V-bottom 96-well plate, with 50 µL of PBS in columns 2–12 and 100 µL of virus in column 1. After performing 2-fold dilutions across the plate, 50 µL of 1% RBC suspension is added to each well. After incubating for 30 min at room temperature, the results are recorded by tilting the plate: a positive result shows uniform RBC agglutination, while a negative result shows tear-drop-shaped RBC settling. The hemagglutination titer is the reciprocal of the highest dilution showing full agglutination. At the end of the experiment, the results of the hemagglutination assay were calculated using the Reed–Muench method.

### 4.4. Construction of shRNA and HA-Tagged Expression Vectors

The shRNA oligos were ordered from Sangong Biotech (Shanghai, China), and the sequences of these oligos are as follows:

ShRNA Scramble - F 5′:

CCGGCAACAAGATGAAGAGCACCAACTCGAGTTGGTGCTCTTCATCTTGTTGTTTTTG

ShRNA Scramble - R 5′:

AATTCAAAAACAACAAGATGAAGAGCACCAACTCGAGTTGGTGCTCTTCATCTTGTTG

ShRNA GARS - F 5′:

CCGGAAGAAGTTGTTCCGAATGTAACTCGAGTTACATTCGGAACAACTTCTTTTTTTG

ShRNA GARS - R 5′:

AATTCAAAAAAAGAAGTTGTTCCGAATGTAACTCGAGTTACATTCGGAACAACTTCTT

ShRNA HARS - F 5′:

CCGGCGAGAAGGTGTTTGACGTAATCTCGAGATTACGTCAAACACCTTCTCG TTTTTG

ShRNA HARS - R 5′:

AATTCAAAAACGAGAAGGTGTTTGACGTAATCTCGAGATTACGTCAAACACCTTCTCG

ShRNA FARSA - F 5′:

CCGGGCGCCCAACGATGATCAAATACTCGAGTATTTGATCATCGTTGGGCGC TTTTTG

ShRNA FARSA - R 5′:

AATTCAAAAAGCGCCCAACGATGATCAAATACTCGAGTATTTGATCATCGTTGGGCGC

The system of the annealing oligos was followed by mixing both 20 μM forward and reverse oligo 5 μL, 5 μL 10 × NEB buffer, and ddH_2_O together (total volume is 50 μL). The program was 95 °C for 4 min, 70 °C for 10 min by PCR machine, and then slowly cooled down to room temperature for at least 2 h. Then, the recombination plasmids (pLKO.1-shGARS and a negative control pLKO.1-shScramble) were generated by restriction digestion of the plasmids with AgeI and EcoRI and ligations with the annealed oligos of indicated genes by T4 ligase at 16 °C overnight. A total of 5 μL of the ligation mix was transformed into 50 μL competent DH5α cells. Then, the successfully ligated plasmids were screened by sequencing.

The full-length cDNA encoding human GlyRS, HARS, or FARSA was cloned into a pcDNA 3.0 vector, which carries a 3× HA tag. The primers used for cloning are as follows, where F1/R1 was used for amplifying the target gene while F2/R2 was used for amplifying the vector:

HA-GlyRS F1:

CCATATGACGTTCCAGATTACGCTatgccctctccgcgtccagtg

HA-GlyRS R1:

CCGCCAGTGTGATGGATATCTtcattcctcgattgtctctttttt

HA-GlyRS F2:

aaaaaagagacaatcgaggaatgaAGATATCCATCACACTGGCGG

HA-GlyRS R2:

cactggacgcggagagggcatAGCGTAATCTGGAACGTCATATGG

HA-HARS F1:

CCATATGACGTTCCAGATTACGCTatggcagagcgtgcggcgctg

HA-HARS R1:

CCGCCAGTGTGATGGATATCTtcagcagatgcagaggggctg

HA-HARS F2:

cagcccctctgcatctgctgaAGATATCCATCACACTGGCGG

HA-HARS R2:

cagcgccgcacgctctgccatAGCGTAATCTGGAACGTCATATGG

HAFARSAF1:

CCATATGACGTTCCAGATTACGCTatggcggatggtcaggtggcg

HA-FARSA R1:

CCGCCAGTGTGATGGATATCTtcacgcagcctcctgtgtggg

HA-FASRA F2:

cccacacaggaggctgcgtgaAGATATCCATCACACTGGCGG

HA-FARSA R2:

cgccacctgaccatccgccatAGCGTAATCTGGAACGTCATATGG

All HA-tagged constructs were constructed using Seamless Clone Construction. 

### 4.5. Lentivirus Assembly and Infection

Two hours prior to the experiment, 293T cells were maintained in antibiotic free medium. High titers of lentivirus were generated by co-transfection of 293T cells with the viral constructs (10 µg) and the packaging plasmids (7.5 µg pMD2.G and 7.5 µg psPAX2) using linear polyethyleneimine (Polysciences Inc., Warrington, PA, USA). Meanwhile, the ShScramble lentivirus was used as the negative control. After transfection for 48 h, the virus-containing supernatants were harvested by centrifugation (1000× *g*, 5 min) and then filtered through a 0.22 µm syringe filter. The A549 cells were infected with the supernatants at a multiplicity of infection of 50, and polybrene was added to a final concentration of 8 µg/mL. Stably transduced cell lines were established by puromycin (5 μg/mL) selection. Discrete colonies resistant to puromycin appeared within a week. The cells were collected for mRNA or protein quantification to confirm knockdown.

### 4.6. Expression and Purification of GlyRS

Human GlyRS was cloned into the pET-21a vector and subsequently transformed into BL21(DE3) cells. Overnight cultures were grown in LB medium supplemented with 100 µg/mL ampicillin until saturation and then diluted 1:100 in fresh LB medium and incubated at 37 °C. Upon reaching an optical density (OD600) of 0.4, isopropyl β-D-thiogalactopyranoside (IPTG) was added to a final concentration of 0.5 mM to induce protein expression. The cells were then incubated at 16 °C for 20 h. Post-induction, the cells were harvested by centrifugation at 4 °C, 4000 rpm for 30 min. The resulting cell pellet was resuspended in lysis buffer (20 mM Tris-HCl, 300 mM NaCl, 5 mM imidazole, 1 mM phenylmethylsulfonyl fluoride (PMSF), pH 8.0). Cell lysis was performed using an ultrasonic cell disruptor, and the lysate was clarified by centrifugation at 4 °C, 20,000 rpm for 1 h. The proteins were purified using Ni-NTA beads (Cytiva, Marlborough, MA, USA), followed by chromatography on a HiTrap Q HP column (Cytiva, Marlborough, MA, USA), and finally, a HiLoad 16/60 Superdex 200 prep grade column (Cytiva, Marlborough, MA, USA). All purification steps were carried out at 4 °C or on ice to maintain protein stability and activity.

### 4.7. Thermal Shift Assay

Thermal shift assays were conducted using a StepOnePlus 7 Flex Real-Time Cycler (Applied Biosystems, Waltham, MA, USA). The Protein Thermal Shift™ Kit dye from Thermo Fisher Scientific was employed to monitor the thermal stability of the protein by binding to its exposed hydrophobic regions. In each well of a 96-well Optical Reaction Plate (Applied Biosystems), a solution containing 14 μL of protein thermal shift buffer, 2 μL of diluted thermal shift dye (8×), 2 μL small molecule solution at 1 mM, and 2 μL of protein at 1 mg/mL was added. The 96-well polymerase chain reaction plates were then placed in the Life Technologies system and incubated at 25 °C for 10 min. Subsequently, the samples were gradually heated from 25 °C to 95 °C at a rate of 1 °C/min. During the thermal denaturation of the protein, the fluorescence signal of SYPRO orange at 490/530  nm excitation/emission wavelengths was recorded by the instrument at 30 s intervals. The fluorescence signal was continuously monitored and plotted against the temperature, with the midpoint of the protein unfolding transition defined as the melting temperature (Tm). To ensure accuracy, triplicate experiments were performed. The ΔTm represents the shift in Tm value between the GlyRS treated with compounds and the blank control.

### 4.8. Bio-Layer Interferometry Assay

Binding experiments on the GlyRS proteins with various molecules were conducted using an Octet R8 system (ForteBio, Fremont, CA, USA). Purified His-tagged GlyRS proteins (500 μg/mL) were captured using Ni-NTA biosensors (ForteBio, Fremont, CA, USA), resulting in a saturation response of 4–5 nm after 300 s. Subsequently, the biosensors were immersed in diluted molecules (0.625–20 μM in assay buffer) for 120 s to measure the association signal, followed by transfer into Octet buffer to measure the dissociation signal for 100 s. Reference wells containing buffer instead of the tested compounds were included to correct for baseline shifts. A parallel set of Ni-NTA sensors immersed only in buffer served as negative reference controls to correct for nonspecific binding of the compounds to the biosensor surface. The signals were analyzed by a double reference subtraction protocol to deduce nonspecific and background signals and signal drifts caused by biosensor variability. All assays were performed by a standard protocol in 96-well black plates with a total volume of 200 μL/well at 30 °C. The generated data were analyzed using the Octet^®^ Analysis Studio 12.2.2.26 for correction and curve fitting.

### 4.9. Enzyme Inhibition Assay

The ATP consumption assay was utilized to assess the enzymatic inhibitory activity of the compounds. The 30 μL reaction mixture consisted of 1 μM GlyRS, 25 μM ATP, 50 μM L-Glycine, 30 mM HEPES pH 7.5, 150 mM NaCl, 30 mM KCl, 40 mM MgCl_2_, and 0.1% bovine serum albumin (BSA), along with the corresponding concentrations of each compound. The reaction mixture was incubated at room temperature for 15 min. After incubation, 10 μL of the reaction reagent was transferred to a 384-well microplate, and the GlyRS enzyme activity was detected by adding 10 μL of the ATP Assay Kit (#S0026, Beyotime, Shanghai, China). Luminescence measurements were performed using a Synergy HTX multimode microplate Reader (BioTek Inc., Winooski, VT, USA). The inhibitory rate was determined based on three independent assays, and the average value was calculated to provide a reliable assessment of the inhibitory activity of each compound.

### 4.10. Molecular Docking

We employed AutoDock Vina version 1.2.2 for molecular docking studies of the GlyRS protein with small molecules [30]. The three-dimensional structure of GlyRS was sourced from the PDB database (PDB ID: 2ZT7), while the small molecules were obtained from the PubChem database. Preparation for docking was performed using AutoDock Tools, which involved merging non-polar hydrogen atoms and assigning Gasteiger charges to generate the receptor PDBQT file. The ligand, N-acetyltyrosine, was similarly prepared and converted to PDBQT format to ensure proper assignment of rotatable bonds and partial charges. The docking grid was centered at coordinates (center_x = −6.524, center_y = 9.433, center_z = 11.551) with dimensions of 50 Å × 50 Å × 50 Å to allow adequate space for ligand exploration. We set the exhaustiveness parameter to 8 and the num_modes parameter to 9, enabling Vina to output up to nine binding poses. Molecular visualization and analysis were performed using Pymol (https://pymol.org, accessed on 25 August 2024).

### 4.11. Molecular Dynamics Simulation

In this study, we examined the interaction between the GlyRS protein and the small molecules Lycobetaine and Scutellarein, forming a protein–small molecule complex. The molecular dynamics (MD) simulations were performed using the Gromacs program. We imported the structural files of the small molecules and proteins into Gromacs. The pdb2gmx and gmx editconf commands were used to generate topological files and define simulation boxes. Structural optimization was achieved using the gmx grompp and gmx mdrun commands, minimizing the energy of the proteins and small molecules to obtain a stable starting configuration. The MD simulations were executed for 100 ns using the gmx grompp and gmx mdrun commands. The simulations were conducted at a constant temperature of 300 K and atmospheric pressure (1 bar), employing the Amber99sb-ildn force field. Tip3p water molecules were used as the solvent. Post-simulation analysis involved calculating the root-mean-square deviation (RMSD) and root-mean-square fluctuation (RMSF) of the protein–small molecule complex using the gmx rms command. The results were visualized through graphs and statistical tables, providing insights into the conformational stability and dynamics of the complex. This methodology enabled a comprehensive analysis of the interactions and dynamics within the GlyRS protein complex with Lycobetaine and Scutellarein.

### 4.12. Western Blot Assay

Total protein was extracted from cells or tissues by the RIPA buffer (50 mM Tris-HCl pH 7.4, 150 mM NaCl, 1% NP40, 1 mM EDTA), supplemented with a protease and phosphatase inhibitor cocktail (Roche). After SDS-PAGE, the protein on the gel was transferred to a PVDF membrane (Bio-Rad, Hercules, CA, USA) and then blocked with 5% nonfat milk in TBST for 1 h. Then, the membranes were incubated with the indicated primary antibodies and corresponding HRP-conjugated secondary antibodies. Signals were detected by the Bio-Rad ChemiDoc system (Bio-Rad, Shanghai, China) and analyzed using ImageJ software. The following antibodies were used: GlyRS antibody (Proteintech, Wuhan, China, 1:3000), GAPDH (Ray antibody biotech, Beijing, China, 1:5000), GAPDH (Ray antibody biotech, Beijing, 1:5000), or beta-actin (Cell Signaling Technology, Boston, MA, USA, 1:1000).

### 4.13. Statistical Analysis

The statistical results of all data in this study were analyzed using SPSS 25 software or GraphPad Prism 9.0 software. Descriptive statistics for numerical variables were presented as mean ± standard deviation (SD) or standard error of the mean (SEM). Differences in means between the two groups were compared using *t*-tests. A significance level of *p* < 0.05 was considered statistically significant.

## Figures and Tables

**Table 1:** The ten positive hits of BLI assay and their K_D_ values on GlyRS.

Compounds	K_D_ (μM)	K_on_ (1/MS) × 10^3^	K_dis_ (1/s) × 10^−2^
Iodoquinol	7.14 ± 0.41	3.10 ± 0.51	2.23 ± 0.49
Chloramine-T	15.09 ± 1.80	52.28 ± 13.31	78.19 ± 17.95
Lansoprazole	12.48 ± 1.63	7.23 ± 2.14	9.25 ± 3.64
Nisoldipine	47.71 ± 7.18	86.16 ± 27.50	400 ± 94.49
Velpatasvir	11.72 ± 1.13	15.80 ± 0.71	18.46 ± 0.98
Mometasone furoate	1.82 ± 0.36	108.8 ± 67.42	31.08 ± 9 03
Carnosic acid	14.54 ± 0.19	1.13 ± 0.26	1.12 ± 0.17
Scutellarein	2 88 ± 0.83	3.96 ± 1.05	1.09 ± 0.11
N-Acetyl-L-alanine	24.85 ± 4.33	396.20 ± 85.48	560 ± 194
Lycobetaine	43.55 ± 16.37	8.48 ± 2 37	39.73 ± 11.18

**Table 2:** The chemical structures, △Tm values, binding affinities to GlyRS, and enzyme activity inhibition on GlyRS of ten small positive hits.

Compounds	Structure	△Tm [°C] ^a^	K_D_ [μM] ^b^	Relative Enzyme Activity [%] ^c^
Iodoquinol	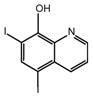	−11.97	7.14 ± 0.41	7.0
Chloramine-T	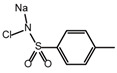	−5.13	15.09 ± 1.90	86.9
Lansoprazole	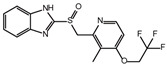	−5.03	12.48 ± 1.63	72.3
Nisoldipine	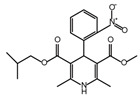	−8.96	47.71 ± 7.18	67.3
Velpatasvir	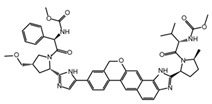	−5.4	11.72 ± 1.13	78.5
Mometasone furoate	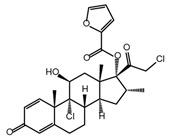	−11.58	1.82 ± 0.36	91.2
Carnosic acid	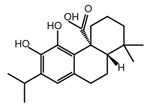	−5.9	14.54 ± 0.19	75.4
Scutellarein	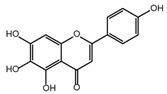	−5.3	2.88 ± 0.83	27.3
N-Acetyl-L-alanine	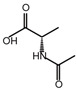	5.96	24.85 ± 4.33	88.1
Lycobetaine	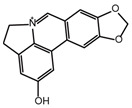	−13.01	43.55 ± 16.37	8.0

^a^ ΔTm is the shift between the Tm values of GlyRS with and without compounds. ^b^ The K_D_ values measured by BLI are presented as the mean ± SD, with the experiment repeated three times. ^c^ Relative enzyme activity represents the ratio of the enzymatic activity of 1 μM GlyRS at 20 μM compounds. The data are the average of two independent experiments.

**Table 3:** List of residues and types of intermolecular interactions involved when GlyRS docked with 10 compounds.

Complex	Docking Score	H-Bond		π-Stacking	Hydrophobic Interactions		Salt Bridges
		Residues	Number	Residues	Residues	Number	Residues
GlyRS- Iodoquinol	−6.88	VAL289	**1**	PHE292			
		ARG529	2				
GlyRS- Chloramine-T	−6.371	ARG529	1	PHE292	VAL289	1	
					PHE292	1	
					ARG529	1	
GlyRS- Lansoprazole	−7.265	GLY406	1	PHE292			GLU403
		ARG529	1				
GlyRS- Nisoldipine	−6.553	GLU245	1		LYS 143	2	LYS143
					PHE144	1	ARG410
GlyRS-Velpatasvir	−7.77	HIS162	1		HIS162	1	
		ARG288	1		LYS165	1	
		ARG529	1		ARG277	1	
					ILE287	1	
					PHE292	2	
					TYR399	1	
GlyRS- Mometasone furoate	−7.627	LYS48	1		ARG49		ARG376
		GLU52	1				
		ILE287	1				
		TYR399	1				
		ILE402	1				
		GLU403	1				
GlyRS- Carnosic acid	−7.646	GLU245	1		PHE144	1	HIS140
		ARG410	1		GLU245	1	LYS143
		GLU522	1				ARG410
GlyRS- Scutellarein	−8.573	ARG277	1		PHE292	1	
		TH293	2		LEU527	1	
		GLU403	2				
		GLY406	1				
		SER524	1				
		GLY528	1				
		ARG529	2				
GlyRS- N-Acetyl-L-alanine	−4.918	VAL289	2		ARG288	1	ARG288
		ARG529	1		VAL289	1	
					PHE292	1	
GlyRS- Lycobetaine	−8.587	VAL289			VAL289	1	
					PHE292	5	

## Data Availability

Data are contained within the article and Supplementary Materials.

## Supplementary Materials

The supporting information can be downloaded at: https://www.mdpi.com/article/10.3390/ijms26072912/s1.
