# Supplementary material for: Glycyl-tRNA Synthetase as a Target for Antiviral Drug Screening Against Influenza Virus
Source: Int J Mol Sci. 2025 Mar 23;26(7):2912. doi: 10.3390/ijms26072912 (PMC11988775; doi:10.3390/ijms26072912)
Supplement: Supplementary file 1 [file ijms-26-02912-s001.zip › Supplementary Figures-3422159-0307.pdf]

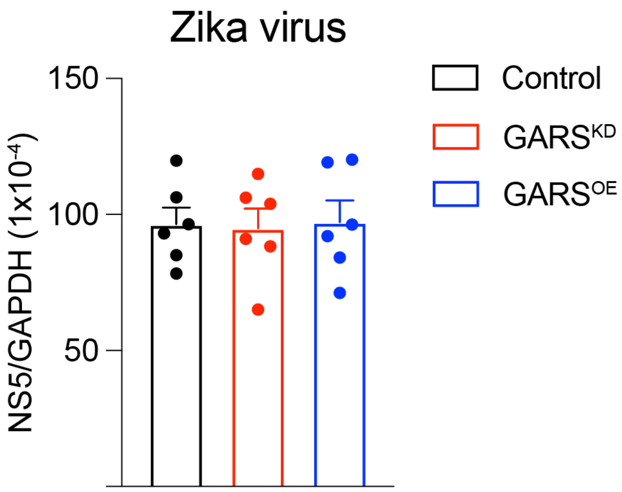

Fig S1. $GARS^{KD}$, $GARS^{OE}$ (transient transfection of HA-GARS) or negative control A549 cells were in-fected with the Zika virus at a MOI of 1. The level of NS5 protein in cells was quantified by qPCR at 48 hours post-infection and normalized to GAPDH.

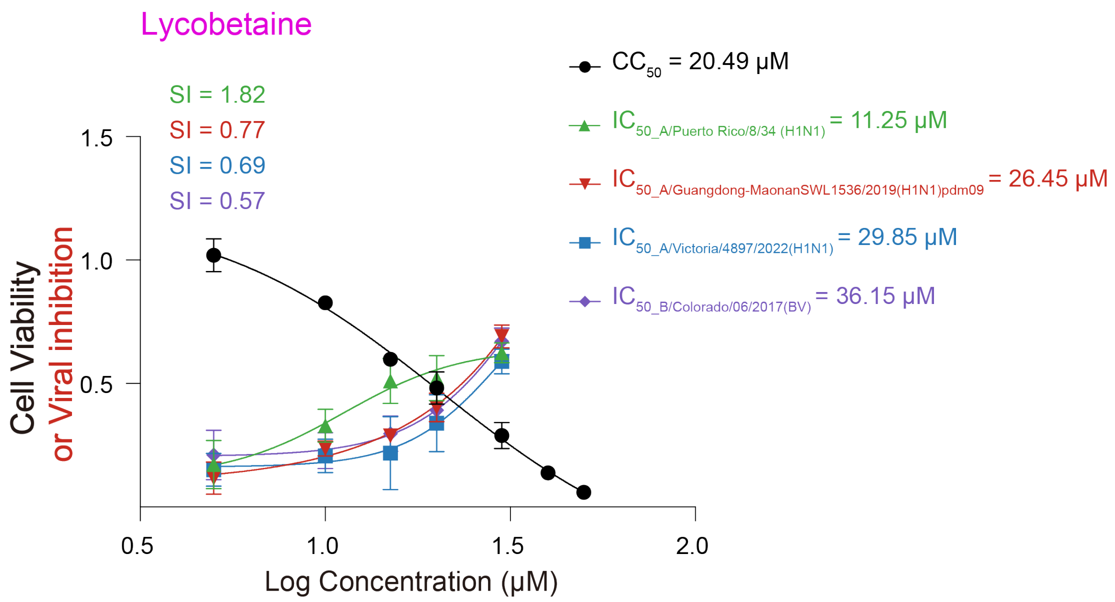

Figure S2. Determination of CC50 and IC50 of Lycobetaine in A549 cells against A/PuertoRico/8/34 (H1N1), A/Guangdong-MaonanSWL1536/2019 (H1N1) pdm09 virus, Victoria/4897/2022(H1N1), and B/Colorado/06/2017(BV). Values of CC50 and IC50 were calculated using non-linear regression analysis with GraphPad Prism 9.0 software.

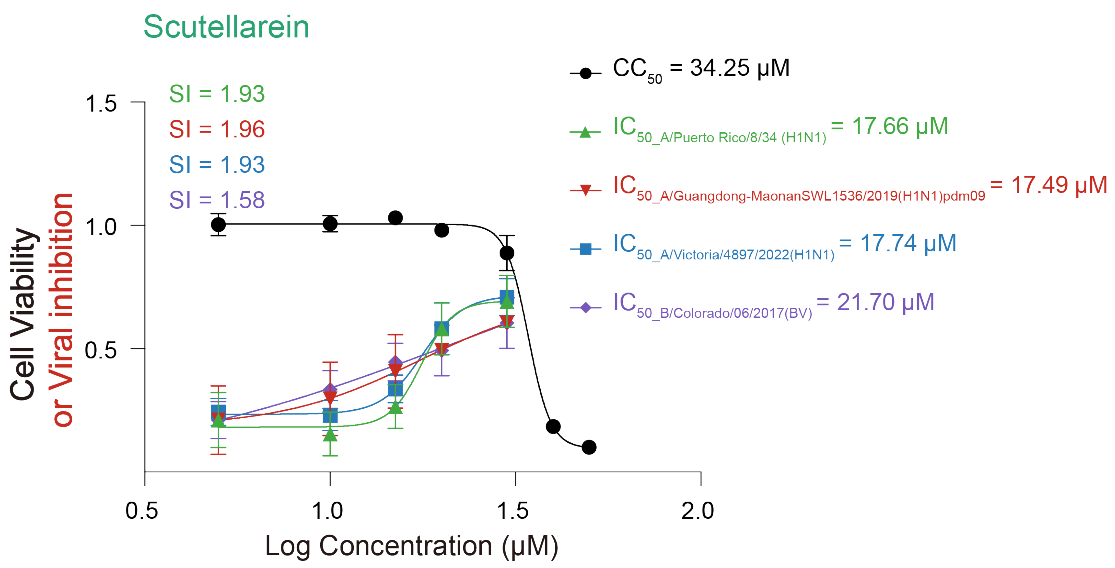

Figure S3. Determination of CC50 and IC50 of Scutellarein in A549 cells against A/PuertoRico/8/34 (H1N1), A/Guangdong-MaonanSWL1536/2019 (H1N1) pdm09 virus, Victoria/4897/2022(H1N1), and B/Colorado/06/2017(BV). Values of CC50 and IC50 were calculated using non-linear regression analysis with GraphPad Prism 9.0 software.

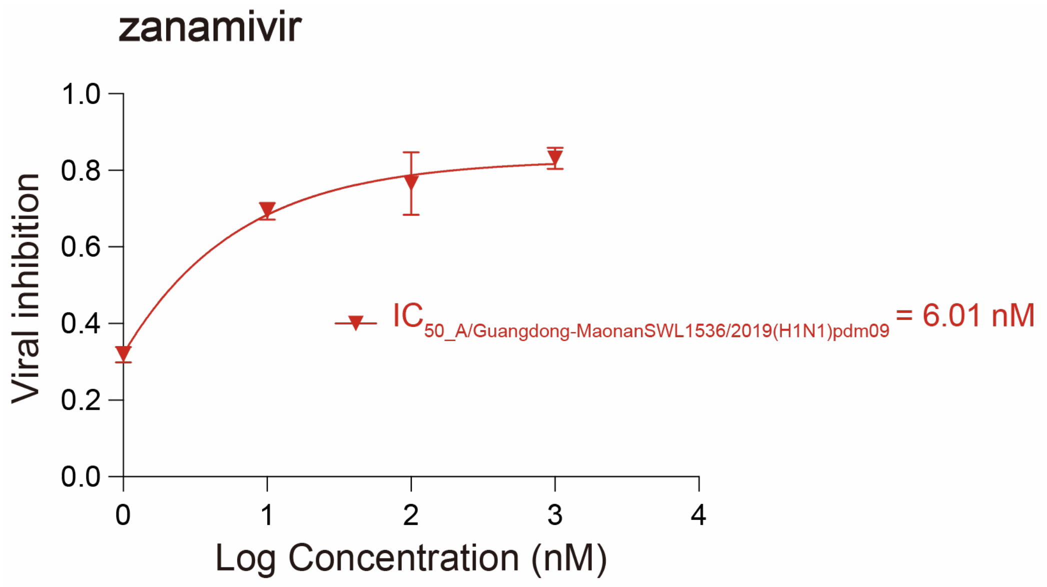

Figure S4. Determination of IC50 of zanamivir in A549 cells against A/Guangdong-MaonanSWL1536/2019 (H1N1) pdm09 virus. Value of IC50 were calculated using non-linear regression analysis with GraphPad Prism 9.0 software.
